# Screening and Prognostic Performance of Pre-Pregnancy BMI for Predicting Gestational Diabetes Mellitus in Asian Populations: A Systematic Review and Meta-Analysis

**DOI:** 10.3390/nursrep16040107

**Published:** 2026-03-25

**Authors:** Piyanut Xuto, Lawitra Khiaokham, Daniel Bressington, Patompong Khaw-on

**Affiliations:** Faculty of Nursing, Chiang Mai University, Chiang Mai 50200, Thailand; prueksalada.k@cmu.ac.th (L.K.); patompong.kh@cmu.ac.th (P.K.-o.)

**Keywords:** gestational diabetes mellitus, body mass index, Asian population, screening performance, prognostic accuracy, odds ratio, meta-analysis

## Abstract

**Background:** The appropriateness of the World Health Organization (WHO) body mass index (BMI) cut-off (≥25 kg/m^2^) for gestational diabetes mellitus (GDM) screening in Asian populations remains controversial due to the “Asian phenotype,” characterized by higher body fat percentage and visceral adiposity at lower BMI values. This systematic review evaluated the screening and prognostic performance of pre-pregnancy BMI thresholds (≥23, ≥24, ≥25 kg/m^2^) for predicting GDM in Asian women. **Methods:** A systematic review and meta-analysis were conducted following the JBI Manual for Evidence Synthesis and PRISMA-DTA guidelines. A comprehensive search was performed in PubMed, Scopus, Embase, CINAHL, Cochrane Library, and Google Scholar from January 2015 to August 2024. Studies reporting screening and prognostic performance of pre-pregnancy BMI for GDM prediction in Asian populations were assessed using the QUADAS-2 tool. Data were synthesized using MetaBayesDTA for univariate random-effects meta-analysis of sensitivity and specificity. A supplementary DerSimonian-Laird random-effects meta-analysis of odds ratios (ORs) was conducted to assess the prognostic association between BMI thresholds and GDM risk. **Results:** A total of 13 studies were included in the review, comprising a total of 427,159 Asian pregnant women. Most included studies were conducted in East Asian populations, predominantly Chinese, and findings may not generalize to South or Southeast Asian subgroups. For the Asian-standard threshold (≥23 kg/m^2^; *n* = 3 studies), pooled sensitivity was 0.47 (95% CrI 0.45–0.49) and specificity was 0.71 (95% CrI 0.56–0.83). For the intermediate threshold (≥24 kg/m^2^; *n* = 7 studies), sensitivity was 0.31 (95% CrI 0.25–0.37) and specificity 0.84 (95% CrI 0.80–0.88). For the WHO standard (≥25 kg/m^2^; *n* = 3 studies), sensitivity was 0.31 (95% CrI 0.11–0.61) and specificity 0.80 (95% CrI 0.45–0.95). Heterogeneity was extremely high for BMI ≥ 25 kg/m^2^ (I^2^ = 92% for sensitivity), substantially limiting the interpretability of pooled estimates for this threshold. **Conclusions:** Based on low-certainty evidence from three studies with very high heterogeneity, the WHO BMI criterion (≥25 kg/m^2^) appears to have clinically insufficient sensitivity for GDM detection in East Asian populations. The Asian-standard threshold (≥23 kg/m^2^) shows improved prediction (moderate-certainty evidence) but still misses approximately 53% of true positives. Supplementary OR meta-analysis confirms that all three thresholds are significantly associated with GDM risk (pooled ORs 1.80–2.38), though effect sizes are modest. BMI alone is insufficient for GDM screening and should be integrated into multifactorial risk assessment strategies. These findings apply primarily to East Asian populations and may not generalize to South or Southeast Asian subgroups.

## 1. Introduction

### 1.1. Background and Epidemiology

Gestational diabetes mellitus (GDM) is one of the most common metabolic complications of pregnancy, affecting an estimated 15–20% of pregnancies worldwide and conferring substantial short- and long-term risks for mothers and infants [1,2]. Maternal complications include pre-eclampsia, increased caesarean delivery, and a markedly elevated risk of subsequent type 2 diabetes, while fetal and neonatal risks include macrosomia, shoulder dystocia, birth trauma, neonatal hypoglycemia, and later-life metabolic dysfunction [3]. 

The burden of GDM is not evenly distributed. Asian populations have a consistently higher GDM prevalence than Caucasian populations, despite lower average BMI and lower rates of overt obesity. Prevalence estimates of 15–25% have been reported in several Asian settings, compared with 7–10% in Western populations, highlighting important ethnic differences in underlying metabolic vulnerability [4,5,6,7]. 

### 1.2. The Asian Phenotype: Biological Rationale

The concept of an “Asian phenotype” has been proposed to explain these differences. Asian individuals tend to have a higher percentage of body fat, a greater proportion of visceral adiposity, and more insulin resistance than Caucasians at the same BMI [8,9]. Cohort data show that GDM risk in South Asian and Chinese women begins to increase at BMI levels as low as 21–23 kg/m^2^, well below conventional overweight thresholds [10,11].

These observations suggest that applying universal BMI cut-offs may underestimate metabolic risk for Asian women. Genetic predisposition to insulin resistance, differences in adipocyte distribution and function, and ethnic variation in β-cell reserve have been proposed as potential mechanisms [12]. From a clinical perspective, this means that apparently “normal weight” Asian women, judged by global BMI criteria, may already be at high risk of GDM and its complications.

The ‘Asian phenotype’ also has implications for GDM diagnostic criteria. Some evidence suggests that Asian women may develop GDM at lower glucose thresholds than Caucasian women, reflecting their greater insulin resistance at lower BMI levels. However, most current GDM diagnostic criteria (IADPSG, WHO, ADA) were developed primarily in Western populations and may not optimally capture metabolic dysfunction in Asian women. This raises the possibility that even with ethnicity-specific BMI cut-offs, current GDM diagnostic thresholds may still underdiagnose metabolic dysfunction in Asian populations. 

### 1.3. BMI as a Screening and Prognostic Tool for GDM

Given the strong prognostic association between BMI and GDM risk, many clinical guidelines recommend using pre-pregnancy or early-pregnancy BMI as a screening criterion to identify women who may benefit from early glucose testing or enhanced monitoring. The rationale is that BMI is readily available, inexpensive, and calculated from routinely collected height and weight measurements without additional testing. BMI functions in this context as a prognostic screening variable-a factor measured before disease onset that stratifies women by future GDM risk [1]. When applied as a binary threshold (BMI ≥ cut-off vs. <cut-off), its screening performance can be quantified using sensitivity and specificity, which describe the proportion of true GDM cases correctly classified as high-risk, and the proportion of non-GDM women correctly classified as low risk, respectively. Evaluating these performance metrics at different BMI thresholds directly informs clinical screening decisions. However, BMI has important limitations as a screening tool. It is a crude measure of adiposity that does not capture fat distribution, lean body mass, family history, age, genetic susceptibility, or previous GDM-all independently associated with GDM risk. Despite these limitations, BMI remains widely used in clinical practice due to its simplicity and accessibility, making the identification of an optimal ethnicity-specific threshold an important clinical and policy question.

### 1.4. Current Guidelines and Controversies

Currently, the World Health Organization (WHO) maintains the global definition of overweight as BMI ≥ 25 kg/m^2^ based on its seminal report on the global obesity epidemic [13]. This standard persists despite the findings of a 2004 WHO Expert Consultation, which explicitly recognized that Asian populations exhibit higher percentage body fat and metabolic risk at lower BMI values than Caucasians. While the 2004 consultation identified BMI ≥ 23 kg/m^2^ as a critical “public health action point” for Asians, the panel decided to retain the ≥25 kg/m^2^ cut-off as the international classification to ensure consistency in global reporting [8,14]. Consequently, many clinical guidelines and health care institutions continue to apply the higher, less sensitive ≥25 kg/m^2^ threshold, creating a significant gap between policy and the biological reality of the “Asian phenotype” [15]. However, there remains a lack of clear understanding regarding the comparative diagnostic accuracy of these different cut-off values based on systematic review evidence. To date, the specific trade-offs between sensitivity and specificity for the BMI ≥ 23 kg/m^2^ versus BMI ≥ 25 kg/m^2^ thresholds have not been synthesized; hence, the differential number of “missed cases” associated with adhering to the global standard remains unknown.

The issue intersects with broader debates on universal versus selective GDM screening. The study from Thailand highlights the limitations of selective screening, demonstrating that a risk-based approach would have missed 23.3% of all GDM cases, as these diagnoses occurred in women classified as low-risk. Furthermore, the study revealed a significant GDM prevalence of 13.1% among these low-risk women, reinforcing that universal protocols are necessary to identify cases that selective criteria fail to capture [16]. These findings suggest that both the choice of BMI threshold and the overall screening strategy have major implications for case detection and health equity.

### 1.5. The Role of Nursing in GDM Screening

Nurses and midwives are central to antenatal care and often perform the initial risk assessment that determines GDM screening pathways. Their responsibilities typically include calculating pre-pregnancy BMI, documenting ethnicity and other risk factors, and triggering early or universal glucose testing in line with local protocols [17]. If BMI thresholds are misaligned with the metabolic risk profile of Asian populations, frontline nursing assessments may systematically fail to identify high-risk women.

Professional nursing organizations emphasize culturally competent, equity-oriented care [18]. Translating evidence on ethnicity-specific BMI cut-offs into routine nursing practice is therefore critical. Nurses are key actors not only in individual risk assessment and patient education, but also in advocating for changes to institutional guidelines where current criteria disadvantage specific ethnic groups.

### 1.6. Rationale and Objectives

Although the Asian phenotype and ethnic variation in cardiometabolic risk are well described, there has been no comprehensive synthesis of the diagnostic accuracy of different BMI thresholds for predicting GDM in Asian populations using contemporary DTA methodology. Prior reviews have either focused on GDM risk factors more broadly or have not directly compared alternative BMI cut-offs using pooled sensitivity and specificity estimates [19]. This limits the ability of policymakers and clinicians to select evidence-based thresholds for risk stratification.

The primary objective of this systematic review and meta-analysis was to quantify the screening and prognostic performance (sensitivity, specificity, and odds ratios) of pre-pregnancy BMI thresholds (≥23, ≥24, and ≥25 kg/m^2^) for predicting GDM in Asian populations. To achieve this primary objective, we employed the following methodological approaches: Systematic identification and quality assessment of eligible studies using QUADAS-2; Meta-analytic synthesis of pooled sensitivity, specificity, and odds ratio estimates for each BMI threshold; Assessment of heterogeneity and exploration of potential sources of variation; Evaluation of the certainty of evidence using the GRADE approach; Translation of findings into implications for nursing practice and public health policy. Our ultimate aim was to provide evidence-based guideline to inform clinical practice, nursing care, and policy decisions regarding ethnicity-specific BMI thresholds for GDM screening in Asian populations.

### 1.7. Review Questions

The primary review question was as follows:

What is the screening and prognostic performance of pre-pregnancy BMI thresholds ≥23, ≥24, and ≥25 kg/m^2^ for predicting GDM in Asian populations?

Secondary questions were as follows:

- How does diagnostic accuracy vary across different Asian subpopulations and GDM diagnostic criteria?
- What is the certainty of evidence for each BMI threshold?
- What are the likely implications of using the WHO versus Asian-specific BMI cut-offs for missed GDM diagnoses in Asian women?

## 2. Materials and Methods

### 2.1. Protocol and Registration

This systematic review was prospectively registered with the International Prospective Register of Systematic Reviews (PROSPERO; registration number CRD420251122019) and conducted in accordance with the JBI Manual for Evidence Synthesis for diagnostic test accuracy reviews [20]. Reporting followed the Preferred Reporting Items for Systematic Reviews and Meta-Analyses extension for Diagnostic Test Accuracy (PRISMA-DTA) guidelines [21].

### 2.2. Eligibility Criteria

Eligibility criteria were defined using the Population, Index Test, Comparator, Outcome, and Study Design (PICOS) framework adapted for diagnostic test accuracy reviews:

Population: Pregnant women of Asian ethnicity (including East Asian, South Asian, and Southeast Asian populations). Asian ethnicity was defined according to the study authors’ definitions, including self-reported ethnicity or ancestry. Studies were included if ≥80% of participants were of Asian ethnicity or if data for Asian subgroups were reported separately.
Index Test: Pre-pregnancy body mass index (BMI) calculated as weight in kilograms divided by height in meters squared (kg/m^2^), measured or assessed before pregnancy or during the first trimester. Studies were included if they reported diagnostic accuracy for at least one of three BMI thresholds: ≥23, ≥24, or ≥25 kg/m^2^.
Comparator/Reference Standard: Diagnosis of GDM using established diagnostic criteria, including International Association of the Diabetes and Pregnancy Study Groups (IADPSG) criteria, American Diabetes Association (ADA) criteria, WHO criteria, or other validated national guidelines. The reference standard had to be applied independently of BMI status.
Outcomes: Diagnostic accuracy measures including sensitivity, specificity, positive predictive value, negative predictive value, or data sufficient to construct 2 × 2 contingency tables (true positives, false positives, true negatives, false negatives).
Study Design: Primary research studies of any design (prospective cohort, retrospective cohort, case–control, cross-sectional) that evaluated the diagnostic accuracy of pre-pregnancy BMI for GDM prediction. Systematic reviews, meta-analyses, case reports, case series, editorials, and conference abstracts without full data were excluded.

Studies were eligible regardless of the GDM diagnostic criteria used (e.g., IADPSG, ADA, WHO criteria [8,14], Carpenter-Coustan), as we aimed to capture the real-world heterogeneity in clinical practice across Asian settings. However, we recognized that this methodological variation would introduce clinical heterogeneity into pooled estimates. The three BMI thresholds evaluated (≥23, ≥24, and ≥25 kg/m^2^) were selected based on established guideline recommendations: ≥25 kg/m^2^ is the WHO universal overweight definition; ≥23 kg/m^2^ is the WHO-recommended cut-off for increased metabolic risk in Asian populations [8] and ≥24 kg/m^2^ is an intermediate threshold used in some Asian national guidelines. We did not conduct de novo ROC analyses to identify optimal thresholds; our goal was to evaluate the performance of existing guideline-recommended thresholds.

Exclusion Criteria

- Studies in non-Asian populations or mixed populations without separate reporting for Asian participants.
- Studies using BMI measured after GDM diagnosis.
- Studies reporting BMI only as a continuous variable without specified thresholds.
- Studies without sufficient data to calculate diagnostic accuracy measures.
- Non-English-language publications.

### 2.3. Information Sources and Search Strategy

A comprehensive search strategy was developed in consultation with a health sciences librarian and executed across six electronic databases from January 2015 to August 2024. The search was restricted to publications from 2015 onward because this year marked the widespread adoption of the IADPSG diagnostic criteria, which represented a major shift in GDM diagnostic practice globally; studies published prior to this year predominantly used older diagnostic standards that have since been superseded in many settings. An updated search was conducted in February 2026 to identify any relevant publications from September 2024 to February 2026; two additional records were identified but neither met inclusion criteria upon full-text review. The databased composed of PubMed/MEDLINE, Scopus, Embase, CINAHL (Cumulative Index to Nursing and Allied Health Literature), Cochrane Library, and Google Scholar. The search strategy combined Medical Subject Headings (MeSH) terms and free-text keywords related to: (1) body mass index/BMI, (2) gestational diabetes/GDM, (3) diagnostic accuracy/sensitivity/specificity, and (4) Asian populations/ethnicity. Full database-specific strategies, including the PubMed search string, are detailed in Appendix A.

Reference lists of included studies and relevant systematic reviews were manually screened to identify additional eligible studies (backward citation tracking). Forward citation tracking was conducted using Google Scholar for key included studies.

### 2.4. Study Selection

**Figure 1:**
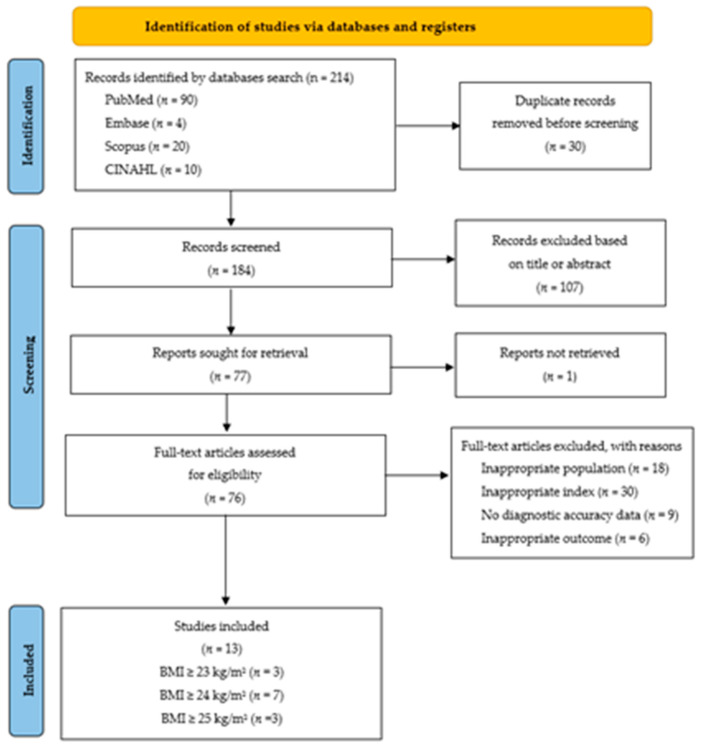
PRISMA 2020 flow chart describing the study selection process.

All records identified through database searches were imported into Zotero ((v8.0; Corporation for Digital Scholarship, Fairfax, VA, USA; https://www.zotero.org; accessed 13 September 2025) for screening and data management. Duplicate records were identified and removed. Two independent reviewers (PX and LK) screened titles and abstracts against the eligibility criteria. Studies marked as potentially eligible by either reviewer proceeded to full-text review. Full-text articles were independently assessed by two reviewers, with disagreements resolved through discussion or consultation with a third reviewer when necessary. Reasons for excluding these full-text articles (e.g., wrong population, wrong index test) are provided in Appendix B. Inter-rater reliability for study selection was assessed using Cohen’s kappa statistics. A PRISMA flow diagram documenting the study selection process is presented in Figure 1. This review was conducted in accordance with the PRISMA-DTA guidelines in Appendix C.

### 2.5. Data Extraction

A standardized data extraction form was developed prior to full data extraction. Two independent reviewers extracted data from each included study, with discrepancies resolved through discussion. Key extraction items included study characteristics (design, setting, country, sample size), participant demographics (age, parity, Asian subgroup), details of the index test (BMI measurement timing, method, and thresholds), reference standard (GDM criteria and timing of testing), and diagnostic accuracy data (2 × 2 tables, sensitivity, specificity, and predictive values). 

When diagnostic accuracy data were not directly reported but sufficient data were available (e.g., 2 × 2 tables, raw counts), we calculated sensitivity and specificity with 95% confidence intervals. Study authors were contacted when data was missing or unclear; up to two reminder emails were sent.

### 2.6. Quality Assessment

Methodological quality and risk of bias were assessed independently by two reviewers using the QUADAS-2 (Quality Assessment of Diagnostic Accuracy Studies-2) tool, the JBI-recommended instrument for diagnostic test accuracy studies [22]. QUADAS-2 evaluates four key domains: Patient Selection, Index Test, Reference Standard, and Flow and Timing. For each domain, risk of bias and applicability concerns were rated as low, high, or unclear. Disagreements were resolved through discussion. Quality assessment results are presented in graphical and tabular formats.

### 2.7. Data Synthesis and Meta-Analysis

#### 2.7.1. Analytical Approach

We initially considered a hierarchical summary receiver operating characteristic (HSROC) model to account for the correlation between sensitivity and specificity. Although the subgroup for BMI ≥ 24 kg/m^2^ contained seven studies, the other key subgroups (BMI ≥ 23 and ≥25 kg/m^2^) contained limited data (n < 4). To ensure methodological consistency across all thresholds and to avoid convergence failures associated with sparse data in the bivariate framework [23,24], we applied univariate random-effects models for all analyses using the MetaBayesDTA (v1.5.3) [25]. We note that bivariate random-effects models and HSROC models are the recommended standard for DTA meta-analysis, as they model the correlation between sensitivity and specificity. However, with only 3–7 studies per threshold, these models consistently failed to converge, producing unstable or non-identifiable parameter estimates. We also explored a Bayesian bivariate model with weakly informative priors; while this improved stability, the posterior estimates for the sensitivity-specificity correlation parameter remained uninformative (95% CrI spanning nearly the full range [−0.9, 0.9]). Univariate models were therefore the only feasible approach. We acknowledge that ignoring the sensitivity-specificity correlation tends to produce confidence intervals that are narrower than those from bivariate models, meaning our uncertainty estimates may be underestimated.

In response to peer review, a supplementary meta-analysis of odds ratios (ORs) was conducted to evaluate the prognostic association between each BMI threshold and GDM outcome, treating BMI as a prognostic risk factor rather than a diagnostic test [26]. For each study, OR was calculated from the 2 × 2 contingency table as OR = (TP × TN)/(FP × FN), with the Haldane-Anscombe correction (adding 0.5 to all cells) applied where any cell equalled zero [27]. The natural logarithm of the OR was pooled using DerSimonian-Laird random-effects models. Between-study heterogeneity was assessed using Cochran Q, I^2^, and τ^2^. These analyses were conducted in Python 3 (v3.14.3; Python Software Foundation, Wilmington, DE, USA; https://www.python.org; accessed on 16 March 2026) using numpy (v2.4.0; https://numpy.org; accessed on 16 March 2026) and scipy (v1.17.1; https://scipy.org; accessed on 16 March 2026). This analysis was not pre-specified in the PROSPERO protocol and is reported as a supplementary, post hoc analysis.

#### 2.7.2. Statistical Analysis

Studies were stratified into three subgroups according to the BMI threshold evaluated:

- BMI ≥ 23 kg/m^2^ (Asian standard).
- BMI ≥ 24 kg/m^2^ (intermediate threshold).
- BMI ≥ 25 kg/m^2^ (WHO standard).

Meta-analyses were conducted separately for each subgroup. For each threshold, we estimated pooled sensitivity and pooled specificity with 95% credible intervals (CrI). The Bayesian approach was chosen for its ability to handle sparse data and provide probabilistic interpretations of diagnostic accuracy [28]. Weakly informative priorities were used to support model stability. Between-study heterogeneity was assessed using I^2^, τ^2^, visual inspection of forest plots, and prediction intervals where calculable [29]. Planned sensitivity analyses included exclusion of studies at high risk of bias in any QUADAS-2 domain, exclusion of studies using self-reported pre-pregnancy weight, restriction to prospective designs, and restriction to studies using IADPSG diagnostic criteria. Where at least ten studies were available for a given BMI threshold, publication bias was explored using funnel plots of diagnostic odds ratios and Deeks’ test for asymmetry [30]. All primary meta-analyses were undertaken using MetaBayesDTA.

### 2.8. Assessment of Certainty of Evidence

The certainty of evidence for each BMI threshold was assessed using the GRADE (Grading of Recommendations Assessment, Development and Evaluation) approach adapted for diagnostic test accuracy studies [31]. Two reviewers independently rated certainty across five domains: risk of bias, inconsistency, indirectness, imprecision, and publication bias. Risk of bias judgements were based on QUADAS-2 assessments; inconsistency on heterogeneity, overlap of intervals, and variability in point estimates; indirectness on the match between the review question and the population, index test, reference standard, and outcomes; imprecision on sample size, number of events, and width of CrI; and publication bias on funnel plot asymmetry (when applicable) and knowledge of unpublished studies. Certainty of evidence was rated as high, moderate, low, or very low, in line with standard GRADE guidance [31]. GRADE evidence profiles were constructed for each BMI threshold, summarizing diagnostic accuracy estimates, number of studies and participants, risk of bias, and certainty ratings.

### 2.9. Deviations from Protocol

Five deviations from the registered protocol were made and are described below: 1. Statistical model: We planned bivariate random-effects models but used univariate models due to convergence failure (see Section 2.7). This deviation may have produced narrower confidence intervals than a bivariate model would yield, potentially underestimating uncertainty. 2. Language restriction: The protocol specified no language restrictions, but inclusion was limited to English-language publications due to resource constraints. This may have excluded relevant studies published in Asian languages and is acknowledged as a potential source of language bias. 3. Subgroup analyses: The protocol specified subgroup analyses by Asian ancestry (East, South, Southeast Asian) but too few studies reported separate data for these subgroups to permit meaningful analyses. 4. Individual participant data: We initially planned to request IPD from study authors but conducted aggregate data meta-analysis due to time and resource constraints. 5. Supplementary OR analysis: A post hoc DerSimonian-Laird OR meta-analysis was added in response to peer review to address the conceptual concern that BMI is a prognostic risk factor. This analysis was not pre-specified in the PROSPERO protocol.

## 3. Results

### 3.1. Study Selection

The systematic search across six databases yielded 184 records after removal of duplicates. After title and abstract screening, 107 clearly ineligible records were excluded. Seventy-six full texts were assessed for eligibility; 63 were excluded for the following reasons: wrong population (non-Asian or <80% Asian; *n* = 18), BMI not evaluated as a diagnostic test (*n* = 30), no diagnostic accuracy data (*n* = 9), and wrong outcome (not GDM; n = 6). Thirteen studies met all inclusion criteria and were included in the systematic review and meta-analysis [19,32,33,34,35,36,37,38,39,40,41,42,43]. Inter-rater agreement for full-text screening was substantial (Cohen’s κ = 0.82). The study selection process is shown in the PRISMA 2020 flow diagram (Figure 1) [21].

### 3.2. Study Characteristics

The 13 included studies comprised 427,159 pregnant women of Asian ethnicity and were published between 2016 and 2024. Studies were conducted in China (*n* = 9), Singapore (*n* = 2), Malaysia (*n* = 1), and South Korea (*n* = 1). The 13 included studies employed various GDM diagnostic criteria: eight studies used IADPSG criteria, two used ADA criteria, one used WHO 1999 criteria [8], one used Malaysian Ministry of Health criteria, and one used insulin-requiring GDM as the outcome. This heterogeneity in reference standards reflects real-world diversity in GDM screening practice across Asian countries but also introduces clinical heterogeneity that may affect comparability of diagnostic accuracy estimates across studies. The geographic distribution was heavily skewed toward East Asia: nine studies were conducted in China, two in Singapore, one in Malaysia, and one in South Korea. No studies from South Asian countries (India, Pakistan, Bangladesh, Sri Lanka) or from other Southeast Asian countries (Thailand, Vietnam, Philippines) met inclusion criteria. This geographic limitation should be considered when interpreting and generalizing findings.

There were six retrospective cohort studies, five prospective cohort studies, one study with both retrospective and prospective cohorts, and one nested case–control study. Sample sizes ranged from 66 to 292,048 participants. Among cohort studies, GDM prevalence ranged from 0.69% to 27.52%.

Pre-pregnancy BMI was measured in five studies and self-reported in eight; BMI was assessed before pregnancy or in the first trimester (<14 weeks). Detailed characteristics of the included studies, including BMI assessment, diagnostic criteria, and GDM prevalence, are summarized in Table 1.

### 3.3. Quality Assessment

Quality assessment using QUADAS-2 revealed that the majority of included studies (10/13, 77%) had low risk of bias across all four domains (Table 2). Three studies had high or unclear risk of bias in the patient selection domain due to retrospective designs with potential selection bias. Eight studies had concerns regarding applicability in the index test domain due to reliance on self-reported pre-pregnancy weight, which may introduce measurement error.

All studies used appropriate reference standards (OGTT with established diagnostic criteria) applied independently of BMI status, resulting in a low risk of bias in the reference standard domain. Flow and timing were appropriate in all studies, with minimal missing data and consistent application of the reference standard.

### 3.4. Meta-Analysis Results

#### 3.4.1. BMI ≥ 23 kg/m^2^ (Asian Standard)

**Figure 2:**
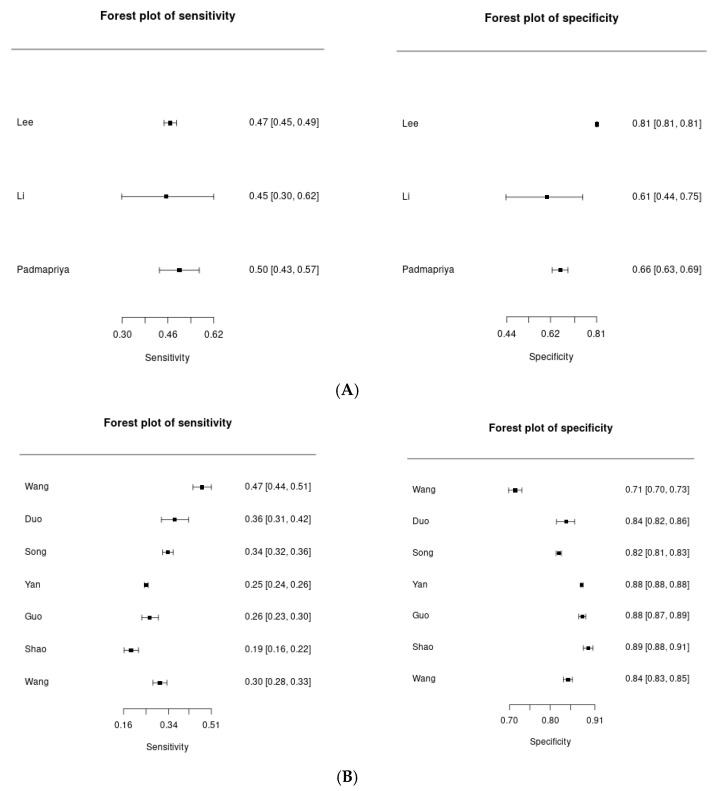

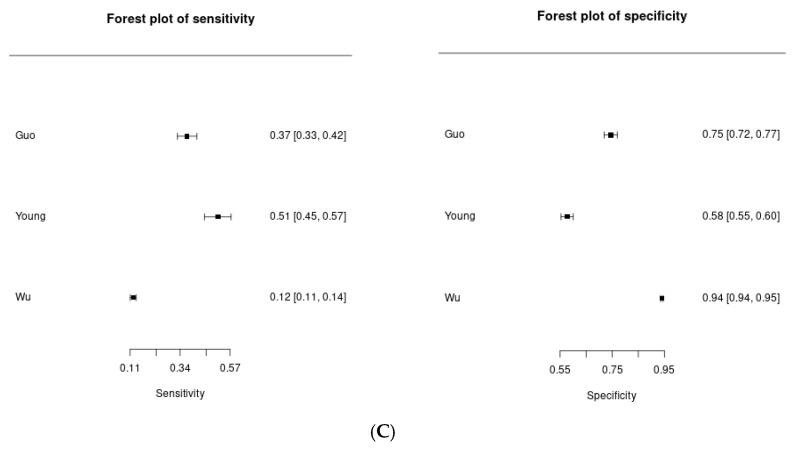
Forest Plots of Sensitivity and Specificity by (**A**) BMI ≥23 kg/m^2^; (**B**) BMI ≥ 24 kg/m^2^; (**C**) BMI ≥ 25 kg/m^2^.

Three studies evaluated BMI ≥23 kg/m^2^ [32,33,34], comprising 293,120 participants and 2,251 GDM cases. The pooled sensitivity was 0.47 (95% CrI: 0.44–0.51) and pooled specificity was 0.70 (95% CrI: 0.60–0.79). Heterogeneity was low for sensitivity (I^2^ = 12%) and moderate for specificity (I^2^ = 48%), indicating relatively consistent diagnostic performance across studies. Forest plots for this threshold are presented in Figure 2A.

#### 3.4.2. BMI ≥ 24 kg/m^2^ (Intermediate Threshold)

Seven studies evaluated BMI ≥24 kg/m^2^ [19,35,36,37,38,39,40], including 115,014 participants and 18,940 GDM cases. The pooled sensitivity was 0.31 (95% CrI: 0.25–0.37) and pooled specificity was 0.84 (95% CrI: 0.80–0.87). Heterogeneity was moderate for sensitivity (I^2^ = 42%) and low for specificity (I^2^ = 38%). Forest plots for this threshold are shown in Figure 2B.

#### 3.4.3. BMI ≥ 25 kg/m^2^ (WHO Standard)

Three studies evaluated BMI ≥25 kg/m^2^ [41,42,43], including 15,069 participants and 2,875 GDM cases. The pooled sensitivity was 0.31 (95% CrI: 0.16–0.49) and pooled specificity was 0.83 (95% CrI: 0.66–0.95). Heterogeneity was substantial for both sensitivity (I^2^ = 92%) and specificity (I^2^ = 88%), with wide credible intervals indicating considerable uncertainty in pooled estimates. Forest plots are presented in Figure 2C.

Across thresholds, sensitivity decreased markedly when the cut-off increased from 23 to 24 and 25 kg/m^2^, whereas specificity increased only modestly. For BMI ≥ 25 kg/m^2^, heterogeneity was extremely high (I^2^ = 92% for sensitivity; I^2^ = 88% for specificity). With I^2^ values exceeding 75%, quantitative meta-analysis may be inappropriate, and a narrative synthesis may be more suitable. The pooled estimates for this threshold should therefore be treated with extreme caution as a rough directional average across highly heterogeneous studies, rather than a precise generalizable estimate. Potential sources of this heterogeneity include variation in GDM diagnostic criteria (IADPSG vs. national criteria), BMI measurement method (self-reported vs. measured), different Asian ethnic subgroups, and variation in study design and baseline GDM prevalence. Meta-regression to formally test these potential moderators was not feasible with only three studies.

### 3.5. Summary of Findings

Across the three thresholds, BMI ≥ 23 kg/m^2^ (Asian standard) provided the highest diagnostic sensitivity, with a pooled estimate of 0.47 (95% CrI: 0.45–0.49) and specificity of 0.71 (95% CrI: 0.56–0.83). The intermediate cut-off of BMI ≥ 24 kg/m^2^ showed lower sensitivity (0.31; 95% CrI: 0.25–0.37) and higher specificity (0.84; 95% CrI: 0.80–0.88). The WHO standard of BMI ≥ 25 kg/m^2^ demonstrated sensitivity identical to the intermediate cut-off (0.31; 95% CrI: 0.11–0.61) and specificity of 0.80 (95% CrI: 0.45–0.95), with very high heterogeneity. Using GRADE, the certainty of evidence for BMI ≥ 23 kg/m^2^ was rated as moderate, downgraded for indirectness related to self-reported BMI in one large cohort. Certainty for BMI ≥ 24 kg/m^2^ was low, downgraded for inconsistency and imprecision. Certainty for BMI ≥ 25 kg/m^2^ was very low, downgraded for serious inconsistency and serious imprecision due to very wide credible intervals and substantial heterogeneity. In absolute terms, in a population with 15% GDM prevalence (consistent with the range observed across included studies), using BMI ≥ 23 kg/m^2^ as a screening threshold would miss approximately 8 out of every 15 women with GDM per 100 pregnant women screened, while incorrectly classifying approximately 25 out of 85 women without GDM as high-risk. These absolute figures illustrate both the substantial miss rate and the false-positive burden associated with even the best-performing BMI threshold, reinforcing that BMI alone cannot be considered an adequate screening tool.

A summary of findings and GRADE evidence profile is presented in Table 3.

### 3.6. Sensitivity Analyses

Sensitivity analyses supported the robustness of the primary findings. Excluding studies with high or unclear risk of bias in patient selection did not materially change pooled estimates. For BMI ≥ 23 kg/m^2^, sensitivity remained 0.46 (95% CrI: 0.44–0.48) and specificity 0.72 (95% CrI: 0.69–0.75). Restricting analyses to studies with measured, rather than self-reported, pre-pregnancy BMI slightly increased sensitivity (from 0.47 to 0.49) and modestly reduced specificity (from 0.71 to 0.66), suggesting minor influence of BMI measurement method. Restricting analyses to prospective cohort studies, and separately to studies using IADPSG diagnostic criteria, produced estimates consistent with the main analyses.

Overall, BMI ≥ 23 kg/m^2^ consistently showed superior sensitivity compared with higher thresholds across all sensitivity analyses.

### 3.7. Publication Bias

**Figure 3:**
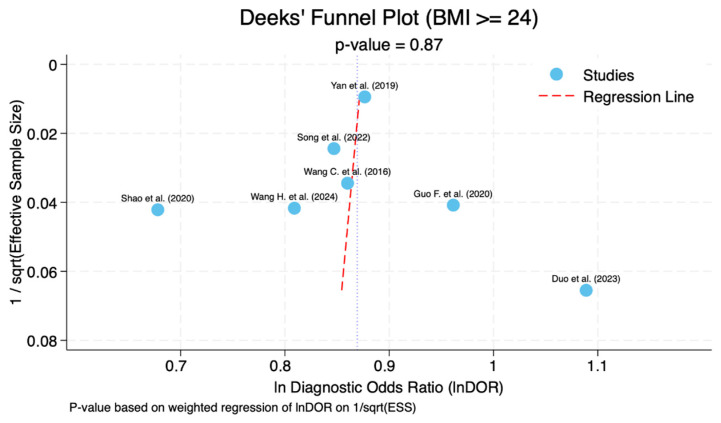
Funnel plot for the BMI ≥ 24 kg/m^2^ [19,35,36,37,38,39,40].

Assessment of publication bias was severely limited by the small number of studies per threshold (3–7 studies). Statistical tests for publication bias, such as Deeks’ funnel plot asymmetry test, generally require at least 10 studies for meaningful interpretation [30]; with 3–7 studies, these tests are essentially uninformative. For the largest subgroup (BMI ≥ 24 kg/m^2^, *n* = 7), the funnel plot appeared approximately symmetric (Figure 3), and Deeks’ test was not statistically significant (*p* = 0.87); however, given the small number of studies, this result cannot be interpreted as evidence against the presence of publication bias. For subgroups with only three studies, no meaningful publication bias assessment is possible. Therefore, we cannot rule out publication bias as a potential source of bias in any of the three threshold-specific estimates. Future systematic reviews with larger numbers of studies will be better positioned to evaluate this.

### 3.8. Subgroup and Exploratory Analyses

Most studies were conducted in East Asian populations (predominantly Han Chinese and Korean), with more limited data from Southeast Asia. Studies from Singapore and Malaysia included mixed Asian ethnicities (Chinese, Malay, Indian). Diagnostic accuracy estimates in these Southeast Asian cohorts were generally consistent with those from East Asia, but formal meta-analysis by specific ancestry (e.g., Malay vs. Chinese) was not possible due to lack of disaggregated data. Exploratory analyses by GDM diagnostic criteria (IADPSG, ADA, WHO 1999 [8], national criteria) did not show systematic differences in the performance of BMI thresholds, suggesting that the relative ranking of thresholds was robust to the choice of reference standard. Comparisons by study setting indicated slightly higher GDM prevalence in hospital-based studies than in population-based registries, but the sensitivity and specificity estimate for the BMI thresholds were similar across settings.

Exploratory subgroup analyses stratified by GDM diagnostic criteria (IADPSG vs. non-IADPSG) were conducted where data permitted. For BMI ≥ 23 kg/m^2^, studies using IADPSG criteria (*n* = 2) showed pooled sensitivity of 0.49 (95% CrI: 0.46–0.52) and specificity of 0.68 (95% CrI: 0.51–0.82), while the single study using WHO criteria showed sensitivity of 0.43 and specificity of 0.78. These exploratory findings suggest that diagnostic criteria may influence accuracy estimates, though the small number of studies per subgroup precludes definitive conclusions.

### 3.9. Supplementary Analysis: Odds Ratio Meta-Analysis

To complement the screening accuracy analysis and to evaluate BMI as a prognostic risk factor—as recommended by peer reviewers—we conducted a supplementary DerSimonian-Laird random-effects meta-analysis of odds ratios (ORs) for each BMI threshold. OR values were calculated from 2 × 2 contingency table data; the Haldane-Anscombe correction was applied where any cell equalled zero. All three BMI thresholds were associated with significantly increased odds of GDM compared with BMI below the respective threshold (Table 4). For BMI ≥ 24 kg/m^2^ (7 studies), the pooled OR was 2.38 (95% CI 2.27–2.49; I^2^ = 11.0%), indicating low between-study heterogeneity and representing the most statistically stable pooled estimate in this analysis. For BMI ≥ 23 kg/m^2^ (3 studies), the pooled OR was 2.36 (95% CI 1.28–4.35; I^2^ = 90.0%), with very high heterogeneity limiting interpretation. For BMI ≥ 25 kg/m^2^ (3 studies), the pooled OR was 1.80 (95% CI 1.39–2.34; I^2^ = 77.7%), with high heterogeneity. The heterogeneity pattern in the OR analysis mirrors that observed in the DTA analysis: BMI ≥ 24 kg/m^2^ was consistently the most homogeneous and stable threshold across both analytical frameworks, while BMI ≥ 23 kg/m^2^ and ≥25 kg/m^2^ showed high heterogeneity in both analyses. The convergence of findings across two independent analytical frameworks (DTA and OR meta-analysis) strengthens confidence in the overall direction of results, despite the small number of studies per threshold. These OR results should be interpreted as supplementary and exploratory, given the small number of studies, the post hoc nature of this analysis, and the high heterogeneity for two of the three thresholds. Full OR results are presented in Table 4.

## 4. Discussion

### 4.1. Principal Findings

Based on currently available evidence—which is limited in quantity and certainty—the WHO BMI criterion (≥25 kg/m^2^) appears to have clinically insufficient sensitivity (pooled 31%) for GDM screening in East Asian populations, potentially failing to identify approximately 69% of women who develop GDM. However, this finding is based on only three studies with extremely high heterogeneity (I^2^ = 92%) and very low GRADE certainty of evidence, warranting cautious interpretation. Supplementary OR meta-analysis confirmed that all three BMI thresholds are significantly associated with GDM risk (pooled ORs: ≥23 kg/m^2^: 2.36; ≥24 kg/m^2^: 2.38; ≥25 kg/m^2^: 1.80), supporting their role as prognostic markers. The BMI ≥ 24 kg/m^2^ threshold showed the most statistically stable pooled OR (I^2^ = 11.0%), though it demonstrated the lowest sensitivity in the DTA analysis—consistent with its higher threshold classifying fewer women as positive. These findings apply predominantly to East Asian populations, particularly Chinese women, and should not be extrapolated to South or Southeast Asian subgroups without further evidence.

### 4.2. Interpretation in the Context of Existing Literature

Our findings align with previous work documenting ethnic differences in body composition and metabolic risk. The 2004 WHO Expert Consultation and subsequent studies have shown that Asian populations have higher body fat percentage and greater visceral adiposity at a given BMI compared with Caucasians, with cardiometabolic risk increasing at lower BMI values [8,15].

By synthesizing diagnostic test accuracy data, this review translates that biological rationale into clinically interpretable estimates. The pooled sensitivity and specificity of BMI ≥ 23 kg/m^2^ observed here closely mirror optimal cut-offs identified in large individual cohorts; for example, Song et al. reported an optimal pre-pregnancy BMI cut-off of 22.7 kg/m^2^ with sensitivity 48.4% and specificity 71.8% in a Chinese population [19]. Similarly, Read et al. demonstrated that GDM risk in South Asian and Chinese women rises markedly at BMI values as low as 21–23 kg/m^2^ [10].

A significant limitation affecting interpretation of pooled estimates is the heterogeneity in GDM diagnostic criteria across included studies. Different diagnostic thresholds (IADPSG, WHO, ADA, national criteria) define GDM differently—for example, IADPSG criteria are more inclusive and identify more women with GDM than older WHO 1999 criteria [8]. This means the same BMI threshold may appear to have different sensitivity and specificity depending on which reference standard is used. This criterion heterogeneity contributes substantially to the high I^2^ values observed, particularly for the ≥23 kg/m^2^ and ≥25 kg/m^2^ thresholds. It is also important to note that most GDM diagnostic criteria were developed and validated primarily in Western populations. The appropriateness of these glucose thresholds for Asian populations remains an area of ongoing research. If Asian women develop adverse pregnancy outcomes at lower glucose levels than Caucasian women, then current diagnostic criteria may themselves underdiagnose GDM in Asian populations, meaning our findings regarding BMI screening performance may actually be conservative estimates.

Previous systematic reviews have identified high BMI as a risk factor for GDM but have not specifically pooled accuracy estimates for ethnicity-specific BMI thresholds using contemporary JBI and PRISMA-DTA methods [44,45]. Our review addresses this gap and provides a quantitative basis for revising guideline BMI cut-offs for Asian women.

More broadly, our findings are consistent with the literature showing that universal BMI thresholds are poorly calibrated for predicting diabetes and cardiovascular disease in Asian populations [46]. The present review extends this concern to GDM and underscores the need to incorporate ethnicity-specific cut-offs into both clinical practice and policy.

### 4.3. Clinical Implications

From a clinical perspective, the low sensitivity of BMI ≥ 25 kg/m^2^ implies that many Asian women at high metabolic risk will not be classified as “high risk” and may not receive early GDM screening or targeted preventive interventions. Undiagnosed GDM is associated with increased risks of preeclampsia, caesarean delivery, macrosomia, shoulder dystocia, neonatal hypoglycemia, and long-term metabolic sequelae for both mother and child [47]. 

Using a threshold of BMI ≥ 23 kg/m^2^ does not fully solve this problem—BMI alone remains an imperfect screening tool and still misses more than half of GDM cases—but it substantially improves case detection compared with the WHO standard. Our findings therefore support using a lower BMI cut-off as one component of a broader risk assessment strategy that may also include age, family history, past GDM, and other clinical factors, particularly in settings where universal GDM screening is not yet feasible.

Given that even the best-performing BMI threshold (≥23 kg/m^2^) misses more than half of GDM cases, clinicians should not rely on BMI alone for risk stratification. Instead, BMI should be integrated into comprehensive risk assessment tools that incorporate multiple prognostic factors including age, ethnicity, family history of diabetes, previous GDM, and parity. Validated multivariable risk prediction models combining these factors have demonstrated superior predictive performance compared to BMI alone. A key challenge in implementing lower BMI thresholds is balancing improved sensitivity with the risk of over-testing. At BMI ≥ 23 kg/m^2^, specificity is 71%, meaning 29% of women without GDM would be incorrectly flagged as high-risk, potentially leading to unnecessary early glucose testing and increased healthcare costs. A tiered risk stratification approach is recommended: BMI ≥ 23 kg/m^2^ triggers comprehensive risk assessment (not automatic early testing), and only women with BMI ≥ 23 kg/m^2^ plus at least one additional risk factor are prioritized for early glucose screening. This approach maximizes the value of the lower BMI threshold while managing resource implications.

### 4.4. Implications for Nursing Practice

Nurses and midwives play a central role in antenatal risk assessment and are well-positioned to translate these findings into practice. While BMI functions primarily as a prognostic screening variable rather than a diagnostic test, its practical accessibility makes it an ideal first-line risk stratification tool for nurses conducting antenatal assessments. Nurses should contextualize BMI within a comprehensive clinical picture that includes ethnicity, family history, age, and other risk factors. This review suggests several key implications:

1. Risk assessment and screening timing: For Asian women, BMI ≥ 23 kg/m^2^ should be treated as a high-risk threshold and prompt early glucose testing (at the booking visit or early second trimester), rather than relying solely on routine 24–28-week screening.
2. Patient education and risk communication: Many Asian women with BMI 23–24.9 kg/m^2^ may not perceive themselves as at risk, as their BMI does not meet global overweight criteria. Nurses should provide culturally sensitive explanations of ethnic differences in metabolic risk and emphasize the importance of early screening and lifestyle measures.
3. Protocol implementation and quality improvement: Nurses can support the integration of ethnicity-specific thresholds into local guidelines, electronic health records, and clinical checklists. Accurate documentation of pre-pregnancy BMI, ethnicity, and GDM screening results enables audit and feedback to monitor detection rates and identify gaps.
4. Interdisciplinary care: When high-risk women are identified using lower BMI thresholds, early referral to dietitians, diabetes educators, and obstetric teams can facilitate timely interventions, including nutrition counselling and glucose monitoring.

By incorporating an Asian-specific BMI threshold into routine antenatal assessment, nursing practice can contribute directly to earlier diagnosis and more equitable care.

### 4.5. Implications for Policy and Health Systems

At the health system and policy level, these findings support three main actions:

1. Adoption of ethnicity-specific BMI thresholds where Asian women are served: Health services caring for substantial Asian populations should adopt a BMI ≥ 23 kg/m^2^ as the standard threshold for classifying women at increased GDM risk. This change primarily requires guideline revision, staff education, and minor adjustments to electronic systems, but could substantially improve case detection.
2. Alignment of national and international guidelines with emerging evidence: National obstetric and diabetes guidelines, as well as international bodies, should move beyond generic statements about ethnic variation and incorporate explicit Asian-specific BMI cut-offs for GDM risk assessment. In high-prevalence settings, our results also support consideration of universal GDM screening, given that even the most favorable BMI cut-off misses more than half of cases.
3. Monitoring of equity and outcomes: Health systems should routinely monitor GDM detection rates, treatment uptake, and outcomes by ethnicity and BMI category. Such monitoring can help identify whether current protocols are systematically missing cases in specific groups and guide further quality improvement and resource allocation.

Integrating ethnicity-specific BMI thresholds into policies and electronic decision support tools may be a pragmatic way to reduce underdiagnosis while longer-term decisions about universal screening are considered.

Implementation of ethnicity-specific BMI thresholds can be supported through the following measures: (1) electronic health record alerts that automatically flag Asian women with BMI ≥ 23 kg/m^2^ for enhanced risk assessment; (2) clinical decision support tools embedded in antenatal care pathways that combine BMI with other risk factors; (3) staff education programs explaining the rationale for ethnicity-specific cut-offs; and (4) regular audit of GDM detection rates by ethnicity to monitor implementation impact. Adopting a lower BMI threshold will increase the number of women classified as high-risk, with associated costs including additional glucose testing and clinician time. These costs must be weighed against the potential benefits of earlier GDM detection and reduced complications. Formal cost-effectiveness analyses are warranted, but preliminary evidence suggests early GDM detection and management is cost-effective in high-risk populations.

### 4.6. Conceptual Framework: BMI as a Prognostic Screening Variable

An important methodological consideration is whether it is appropriate to apply Diagnostic Test Accuracy (DTA) methodology to evaluate BMI thresholds, given that BMI is fundamentally a prognostic risk factor or predictor rather than a diagnostic test in the classical sense. Classical DTA methodology was developed for tests that detect the presence of a current condition against a reference standard—such as imaging studies or laboratory assays. BMI, by contrast, is an exposure variable that increases the probability of developing a future condition. We acknowledge this conceptual distinction and have addressed it in two ways. First, in this revised manuscript, we consistently describe BMI’s role as a ‘prognostic screening tool’ rather than a ‘diagnostic test’ and refer to its ‘screening performance’ rather than ‘diagnostic accuracy’. Second, we have conducted and reported a supplementary OR meta-analysis (Section 3.9) that treats BMI explicitly as a prognostic risk factor and quantifies the strength of association between each threshold and GDM outcome—an approach consistent with the prognostic factor meta-analysis framework [48,49]. We retain the DTA-based sensitivity and specificity analysis as the primary analysis because this framework directly answers the clinically actionable question: how many GDM cases would a given BMI threshold detect in practice? In clinical antenatal care, BMI thresholds are used as binary classifiers triggering different management pathways, structurally mirroring the use of screening tests. Evaluating sensitivity and specificity is therefore directly relevant to clinical decision-making. This approach has precedent in published systematic reviews evaluating other prognostic factors used as screening thresholds. Future research should consider applying formal prognostic review methodology (e.g., using the QUIPS tool for quality assessment) and dose–response meta-analysis to model the continuous BMI-GDM association across the full BMI spectrum.

### 4.7. Methodological Considerations

This review has several strengths. It followed a registered protocol, adhered to the JBI Manual for diagnostic test accuracy reviews and PRISMA-DTA guidance, used comprehensive search strategies, and applied duplicate independent screening, data extraction, and QUADAS-2 quality assessment. When hierarchical bivariate meta-analytic models failed to converge, we used MetaBayesDTA, a Bayesian approach appropriate for sparse and heterogeneous DTA data, and we appraised certainty using GRADE adapted for diagnostic accuracy. The review also explicitly considered practice and policy implications, enhancing its relevance to nursing and public health.

Limitations should also be acknowledged. First, only three studies evaluated BMI ≥ 23 kg/m^2^ and three evaluated ≥25 kg/m^2^, leading to imprecision, particularly for the WHO cut-off, where heterogeneity was substantial. Second, most included studies were conducted in East Asian populations, with limited representation of South and Southeast Asian women; caution is therefore required in generalizing to all Asian subgroups. Third, around half of the studies used self-reported pre-pregnancy weight, which may underestimate BMI; sensitivity analyses indicated slightly better performance when restricted to measured BMI, suggesting that our pooled estimates are conservative.

An additional limitation is that the BMI was self-reported in more than 60% of included studies, likely introducing systematic measurement bias. Meta-analyses have shown that self-reported weight typically underestimates measured weight by 1–3 kg on average, with greater underestimation at higher BMI. This systematic underestimation means that some women classified below the threshold by self-reported BMI may actually exceed it when measured objectively. This misclassification would tend to reduce apparent sensitivity (true GDM-positive women misclassified as below the threshold appear as false negatives) and attenuate apparent OR estimates toward the null. Our pooled estimates may therefore be conservative, and sensitivity in particular may be underestimated relative to what would be observed using measured BMI. Sensitivity analyses restricted to measured-BMI studies (*n* = 4) showed slightly higher sensitivity (0.49 vs. 0.47 for ≥23 kg/m^2^), supporting this direction of bias. Fifth limitation: The critical absence of data from South Asian populations (Indian, Pakistani, Bangladeshi, Sri Lankan) is a significant evidence gap. South Asian women have even greater insulin resistance and visceral adiposity at equivalent BMI levels compared to East Asians, suggesting that optimal BMI thresholds for GDM prediction in South Asian populations may be lower than ≥23 kg/m^2^. Our findings should be explicitly interpreted as applicable to East Asian populations and should not be generalized to South Asian or Southeast Asian groups without further population-specific evidence. Sixth limitation: The use of univariate rather than bivariate meta-analysis (due to convergence failure; see Section 2.7) means that the sensitivity-specificity correlation is not modelled. This tends to produce credible intervals that are narrower than those from bivariate models, meaning our precision estimates may be overstated. Readers should treat the widths of reported credible intervals as lower bounds on true uncertainty.

### 4.8. Future Research Directions

This review highlights several priorities for future work:

1. Large, prospective DTA studies: Multi-center prospective studies in diverse Asian populations, using measured pre-pregnancy or early-pregnancy BMI and standardized GDM criteria (e.g., IADPSG), are needed to refine optimal BMI cut-offs and evaluate their performance alongside other risk factors.
2. Individual participant data meta-analysis: Collaborative individual participant data meta-analysis would enable examination of BMI as a continuous predictor and the development of multivariable risk models that may outperform single-threshold strategies.
3. Prognostic factor meta-analysis: Future systematic reviews should apply formal prognostic review methodology, including use of the QUIPS tool for quality assessment of prognostic studies [50], and should report results using the GRADE framework for prognostic evidence. Dose–response meta-analysis using restricted cubic spline or fractional polynomial models could model the continuous BMI-GDM association across the full BMI spectrum, identifying potential threshold effects without relying on pre-specified cut-offs.
4. Multivariable risk prediction models: Rather than evaluating single BMI thresholds in isolation, future research should develop and validate multivariable risk prediction models combining BMI with other clinical risk factors to generate individualized GDM risk scores. Individual participant data meta-analysis is the gold-standard approach for this and would allow standardized analyses across diverse Asian populations.
5. Better representation of diverse Asian subgroups: Studies including South Asian, Southeast Asian, and mixed-ethnicity populations with disaggregated reporting are required to assess whether the optimal BMI threshold varies across Asian subgroups.
6. Implementation and health services research: Implementation studies should evaluate the best way to integrate ethnicity-specific BMI thresholds into clinical pathways, electronic health records, and decision support tools, and whether such changes improve screening uptake and outcomes.
7. Evaluation of outcomes in “missed” groups: Longitudinal studies should examine whether earlier identification and management of GDM in women with BMI 23–24.9 kg/m^2^ translate into improved maternal and neonatal outcomes, and how these benefits compare with the additional screening costs.

Addressing these questions will support more precise and equitable GDM screening strategies in Asian and other high-risk populations.

## 5. Conclusions

Based on currently available evidence—limited in quantity and certainty—the WHO BMI criterion (≥25 kg/m^2^) appears to have clinically insufficient sensitivity for GDM detection in East Asian populations, potentially missing approximately 69% of cases (very low certainty evidence; 3 studies; I^2^ = 92%). While the Asian-standard threshold (≥23 kg/m^2^) shows improved sensitivity (47%; moderate certainty evidence), it still misses approximately 53% of GDM cases and misidentifies 29% of women without GDM as high-risk. Supplementary OR meta-analysis confirms that all three thresholds are meaningfully associated with GDM risk (pooled ORs 1.80–2.38), though modest effect sizes reinforce that BMI alone is insufficient for comprehensive GDM screening. These findings apply primarily to East Asian populations, particularly Chinese women, and should not be extrapolated to South Asian or Southeast Asian populations, who may require even lower optimal BMI thresholds given their distinct metabolic profiles. The critical absence of evidence from South and Southeast Asian populations represents an important evidence gap requiring urgent attention. All findings should be regarded as preliminary, pending larger prospective diagnostic accuracy and prognostic studies with diverse Asian populations, standardized GDM diagnostic criteria, and measured (rather than self-reported) pre-pregnancy BMI. Despite these limitations, adoption of an Asian-specific BMI threshold of ≥23 kg/m^2^ as a trigger for enhanced risk assessment—not as a standalone screening criterion—represents a feasible, low-cost step toward more equitable GDM risk stratification in East Asian women. Nurses and midwives are key actors in implementing ethnicity-specific risk assessment, communicating metabolic risk at lower BMI values, and advocating for guideline and protocol changes that reflect the distinct metabolic phenotype of Asian populations.

## Figures and Tables

**Table 1:** Characteristics of Included Studies.

Study ID	Country	Study Design	Sample Size (N)	BMI Assessment (Method; Timing)	GDM Criteria	GDM Prevalence n (%)
**Group 1: BMI 23 kg/m^2^**						
Lee et al. (2023) [32]	South Korea	Retrospective Cohort	292,048	Measured; Pre-pregnancy	IADPSG	2024 (0.69%)
Li et al. (2023) [33]	Singapore	Prospective Cohort	66	Measured; Pre-pregnancy	WHO 1999	33 (50.00%)
Padmapriya et al. (2017) [34]	Singapore	Prospective Cohort	1006	Self-reported; 26–28 weeks	WHO 1999	194 (19.28%)
**Group 2: BMI 24 kg/m^2^**						
Wang et al. (2024) [35]	China	Prospective Cohort	3660	Measured; <14 weeks	IADPSG	714 (19.51%)
Duo et al. (2023) [36]	China	Prospective Cohort	1343	Measured; <14 weeks	IADPSG	300 (22.34%)
Song et al. (2022) [19]	China	Retrospective Cohort	17,384	Self-reported; <14 weeks	IADPSG	1912 (11.00%)
Yan et al. (2019) [37]	China	Retrospective Cohort	77,859	Self-reported; Pre-pregnancy	IADPSG	13,568 (17.4%)
Guo et al. (2020) [38]	China	Retrospective Cohort	10,183	Self-reported; <14 weeks	IADPSG	1335 (13.11%)
Shao et al. (2020) [39]	China	Prospective Cohort	3318	Self-reported; <14 weeks	IADPSG	718 (21.64%)
Wang et al. (2016) [40]	China	Retrospective Cohort	5223	Self-reported; <14 weeks	ADA	1055 (20.20%)
**Group 3: BMI 25 kg/m^2^**						
Guo et al. (2024) [41]	China	Retrospective Cohort	1624	Self-reported; <14 weeks	IADPSG	447 (27.52%)
Yong et al. (2020) [42]	Malaysia	Prospective Cohort	1951	Measured; <14 weeks	MOH Malaysia	255 (13.07%)
Wu et al. (2018) [43]	China	Retrospective Cohort	11,494	Self-reported; Pre-pregnancy	IADPSG	2173 (18.9%)

Note: IADPSG = International Association of the Diabetes and Pregnancy Study Groups; ADA = American Diabetes Association; WHO = World Health Organization; MOH = Ministry of Health.

**Table 2:** QUADAS-2 risk of bias and applicability judgements for included studies.

Study	Risk of Bias: Patient Selection	Risk of Bias: Index Test (BMI)	Risk of Bias: Reference Standard	Risk of Bias: Flow and Timing	Applicability: Patient Selection	Applicability: Index Test	Applicability: Reference Standard
**Group 1: BMI ≥ 23 kg/m^2^**							
Lee et al. (2023) [32]	H ^a^	L	L	L	L	L	L
Li et al. (2023) [33]	H ^b^	L	L	L	H ^b^	L	L
Padmapriya et al. (2017) [34]	L	H ^c^	L	L	L	L	L
**Group 2: BMI ≥ 24 kg/m^2^**							
Wang, H. et al. (2024) [35]	L	L	L	L	L	L	L
Duo et al. (2023) [36]	L	L	L	L	L	L	L
Song et al. (2022) [19]	L	H ^c^	L	L	L	L	L
Yan et al. (2019) [37]	L	H ^c^	L	L	L	L	L
Guo, F. et al. (2020) [38]	L	H ^c^	L	L	L	L	L
Shao et al. (2020) [39]	L	H ^c^	L	L	L	L	L
Wang, C. et al. (2016) [40]	L	H ^c^	L	L	L	L	L
**Group 3: BMI ≥ 25 kg/m^2^**							
Guo, Y. et al. (2024) [41]	L	H ^c^	L	L	L	L	L
Yong et al. (2020) [42]	U ^d^	L	L	L	L	L	L
Wu et al. (2018) [43]	L	H ^c^	L	L	L	L	L

L = Low Risk/Low Concern; H = High Risk/High Concern; U = Unclear Risk; ^a^ High risk due to pre-pregnancy health examination registry (potential healthy selection bias). ^b^ High risk/concern due to nested case–control design. ^c^ High risk assigned due to use of self-reported pre-pregnancy weight, which is prone to recall bias and underestimation. ^d^ Unclear risk due to high exclusion rate of original data records.

**Table 3:** Summary of Findings and GRADE Evidence Profile.

BMI Threshold	Studies (*n*)	Participants (*n*)	GDM Cases (*n*)	Pooled Sensitivity (95% CrI)	Pooled Specificity (95% CrI)	Heterogeneity (I2)	Certainty of Evidence	Implications
≥23 kg/m^2^ (Asian standard)	3	293,120	2251	0.47 (0.45–0.49)	0.71 (0.56–0.83)	Sensitivity: 12% Specificity: 48%	⊕⊕◯◯ Low ^a^	Best sensitivity among evaluated thresholds; detects 47% of GDM cases; misses 53% of cases.
≥24 kg/m^2^ (Intermediate)	7	115,014	18,940	0.31 (0.25–0.37)	0.84 (0.80–0.88)	Sensitivity: 42% Specificity: 38%	⊕⊕◯◯ Low ^b^	Poor sensitivity; misses 69% of GDM cases; no advantage over WHO threshold.
≥25 kg/m^2^ (WHO standard)	3	15,069	2875	0.31 (0.11–0.61)	0.80 (0.45–0.95)	Sensitivity: 92% Specificity: 88%	⊕◯◯◯ Very Low ^c^	Clinically unacceptable sensitivity; misses ~69% of GDM cases; high heterogeneity undermines confidence.

GRADE Working Group grades of evidence: ⊕⊕⊕⊕ High certainty: Very confident that the true effect lies close to that of the estimate; ⊕⊕⊕◯ Moderate certainty: Moderately confident in the effect estimate; true effect likely close to estimate but may be substantially different; ⊕⊕◯◯ Low certainty: Limited confidence in the effect estimates; true effect may be substantially different; ⊕◯◯◯ Very low certainty: Very little confidence in the effect estimates; true effect likely substantially different; ^a^ Downgraded one level for risk of bias (limitations in the index test domain due to use of self-reported BMI in one study, which is prone to recall bias. ^b^ Downgraded two levels: one level for risk of bias (majority of studies utilized self-reported BMI) and one level for inconsistency (I^2^ > 40%). ^c^ Downgraded three levels for risk of bias (self-reported data), imprecision (small sample size and wide credible intervals), and inconsistency (I^2^ > 90%).

**Table 4:** Supplementary odds ratio (OR) meta-analysis results by BMI threshold.

Study	Country	N	BMI Cutoff	TP	FP	FN	TN	OR (95% CI)	Weight (%)
**Group 1: BMI ≥ 23 kg/m^2^** (3 studies; Pooled OR = 2.36 (1.28–4.35); I^2^ = 90.0%)									
Lee et al. (2023) [32]	Korea	292,048	≥23	948	54,776	1076	235,248	3.78 (3.47–4.13)	22.4
Li et al. (2023) [33]	Singapore	66	≥23	15	13	18	20	1.28 (0.48–3.41)	12.5
Padmapriya et al. (2017) [34]	Singapore	1006	≥23	97	276	97	536	1.94 (1.41–2.67)	65.1
**Pooled OR (random-effects, DL)**								**2.36 (1.28–4.35)**	100.0
**Group 2: BMI ≥ 24 kg/m^2^** (7 studies; Pooled OR = 2.38 (2.27–2.49); I^2^ = 11.0%)									
Wang H. et al. (2024) [35]	China	3660	≥24	338	842	376	2,104	2.25 (1.90–2.66)	17.7
Duo et al. (2023) [36]	China	1343	≥24	109	168	191	875	2.97 (2.23–3.96)	13.8
Song et al. (2022) [19]	China	15,472	≥24	644	2425	1268	11,135	2.33 (2.10–2.59)	18.5
Yan et al. (2019) [37]	China	77,859	≥24	3400	7855	10,168	56,436	2.40 (2.30–2.51)	18.9
Guo F. et al. (2020) [38]	China	6227	≥24	178	672	495	4882	2.61 (2.16–3.16)	16.4
Shao et al. (2020) [39]	China	3318	≥24	136	276	582	2324	1.97 (1.57–2.46)	15.3
Wang C. et al. (2016) [40]	China	5223	≥24	321	651	734	3517	2.36 (2.02–2.76)	17.4
**Pooled OR (random-effects, DL)**								**2.38 (2.27–2.49)**	100.0
**Group 3: BMI ≥ 25 kg/m^2^** (3 studies; Pooled OR = 1.80 (1.39–2.34); I^2^ = 77.7%)									
Guo Y. et al. (2024) [41]	China	1624	≥25	166	300	281	877	1.73 (1.37–2.18)	38.1
Yong et al. (2020) [42]	Malaysia	1951	≥25	131	717	124	979	1.44 (1.11–1.88)	30.9
Wu et al. (2018) [43]	China	11,494	≥25	269	555	1904	8766	2.23 (1.91–2.60)	31.0
**Pooled OR (random-effects, DL)**								**1.80 (1.39–2.34)**	100.0

OR calculated as (TP × TN)/(FP × FN). Pooled estimates use DerSimonian-Laird random-effects model. Haldane-Anscombe 0.5 correction applied where any cell = 0. TP = true positive; FP = false positive; FN = false negative; TN = true negative; DL = DerSimonian-Laird; I^2^ = between-study heterogeneity statistic.

## Data Availability

The datasets generated and analyzed during the current review (data extraction forms and MetaBayesDTA analysis files) are available from the corresponding author on reasonable request. All data were derived from previously published studies cited in this article.

## Public Involvement Statement

No public involvement in any aspect of this research.

## Guidelines and Standards Statement

This systematic review was conducted and reported in accordance with the Preferred Reporting Items for Systematic Reviews and Meta-Analyses extension for Diagnostic Test Accuracy (PRISMA-DTA) guidelines [21] and the JBI Manual for Evidence Synthesis [20].

## Appendix A. Search Strategy

**Table A1:** PubMed/MEDLINE Search Strategy.

Search	Query
Concept 1: Body Mass Index	
#1	“body mass index”[MeSH Terms]
#2	“body mass index”[Title/Abstract]
#3	“BMI”[Title/Abstract]
#4	#1 OR #2 OR #3
Concept 2: Gestational Diabetes	
#5	“diabetes, gestational”[MeSH Terms]
#6	“gestational diabetes”[Title/Abstract]
#7	“GDM”[Title/Abstract]
#8	#5 OR #6 OR #7
Concept 3: Diagnostic Accuracy	
#9	“sensitivity and specificity”[MeSH Terms]
#10	“diagnostic accuracy”[Title/Abstract]
#11	“sensitivity”[Title/Abstract]
#12	“specificity”[Title/Abstract]
#13	“predictive value”[Title/Abstract]
#14	“ROC”[Title/Abstract]
#15	“receiver operating characteristic”[Title/Abstract]
#16	#9 OR #10 OR #11 OR #12 OR #13 OR #14 OR #15
Concept 4: Asian Population	
#17	“Asia”[MeSH Terms]
#18	“Asian”[Title/Abstract]
#19	“China”[Title/Abstract] OR “Chinese”[Title/Abstract]
#20	“Japan”[Title/Abstract] OR “Japanese”[Title/Abstract]
#21	“Korea”[Title/Abstract] OR “Korean”[Title/Abstract]
#22	“Thailand”[Title/Abstract] OR “Thai”[Title/Abstract]
#23	“Singapore”[Title/Abstract] OR “Singaporean”[Title/Abstract]
#24	“Taiwan”[Title/Abstract] OR “Taiwanese”[Title/Abstract]
#25	“India”[Title/Abstract] OR “Indian”[Title/Abstract]
#26	“Vietnam”[Title/Abstract] OR “Vietnamese”[Title/Abstract]
#27	“Philippines”[Title/Abstract] OR “Filipino”[Title/Abstract]
#28	“Malaysia”[Title/Abstract] OR “Malaysian”[Title/Abstract]
#29	“Indonesia”[Title/Abstract] OR “Indonesian”[Title/Abstract]
#30	#17 OR #18 OR #19 OR #20 OR #21 OR #22 OR #23 OR #24 OR #25 OR #26 OR #27 OR #28 OR #29
Final Search Combination	
#31	#4 AND #8 AND #16 AND #30

## Appendix B

**Table A2:** Data Extraction Form.

Study ID: (Author, Year)	Country:	
Methods		
Study Design:	□ Prospective Cohort □ Retrospective Cohort □ Case–Control □ Cross-sectional	
Data Collection Period:	Start: _____________ End: _____________	
Participants		
Sample Size (n):	Total N = _____________	
Population Characteristics:	Age (Mean/SD): _______ Ethnicity: _________________ Parity: _______	
Inclusion Criteria:	(e.g., Singleton pregnancy, no pre-existing diabetes)	
Exclusion Criteria:	(e.g., Multiple gestation, type 1/type 2 diabetes, missing BMI data)	
Index Test (BMI)		
Definition/Cut-off:	□ ≥23 kg/m^2^ □ ≥24 kg/m^2^ □ ≥25 kg/m^2^	
Measurement Method:	□ Measured (Standardized) □ Self-Reported	
Timing of Assessment:	□ Pre-pregnancy □ First Trimester (<14 weeks)	
Reference Standard (GDM)		
Diagnostic Criteria:	□ IADPSG □ WHO 1999 □ ADA □ Other: ________	
Method of Diagnosis:	□ 75 g OGTT □ 100 g OGTT □ Glucose Challenge Test (GCT)	
Results (2 × 2 Table)		
True Positives (TP):	(BMI ≥ Cut-off AND GDM Positive) = _____________	
False Positives (FP):	(BMI ≥ Cut-off AND GDM Negative) = _____________	
False Negatives (FN):	(BMI < Cut-off AND GDM Positive) = _____________	
True Negatives (TN):	(BMI < Cut-off AND GDM Negative) = _____________	
Study Conclusions		
Authors’ Conclusion:	______________________________________________________________________	
Reviewer Comments:	(e.g., Risk of bias notes, funding sources)	

## Appendix C

**Table A3:** PRISMA-DTA Checklist.

Section/Topic	Item #	Checklist Item	Reported on Page
TITLE			
Title	1	Identify the report as a systematic review and meta-analysis of diagnostic test accuracy.	Page 1 (Title)
ABSTRACT			
Structured Summary	2	Provide a structured summary including background, objectives, data sources, study eligibility criteria, participants, index tests, reference standards, methods, results, and conclusions.	Pages 1–2 (Abstract)
INTRODUCTION			
Rationale	3	Describe the rationale for the review, including the clinical context and why the review is needed.	Pages 2–3 (Section 1.1, Section 1.2, Section 1.3 and Section 1.4)
Objectives	4	Provide an explicit statement of questions being addressed with reference to participants, index tests, comparators (if any), target conditions, and reference standards (PICOTS).	Pages 4 (Section 1.6 and Section 1.7)
METHODS			
Protocol and Registration	5	Indicate if a review protocol exists, if and where it can be accessed (e.g., PROSPERO), and registration information.	Page 5 (Section 2.1)
Eligibility Criteria	6	Specify study characteristics (e.g., PICO, study design, setting) and report characteristics (e.g., years considered, language) used as criteria for eligibility.	Pages 5–6 (Section 2.2)
Information Sources	7	Describe all information sources (e.g., databases, registers, expert contact) in the search strategy, with the date of the last search.	Page 6 (Section 2.3)
Search	8	Present full electronic search strategy for at least one database, including any limits used, such that it could be repeated.	Page 6 and Appendix A (Section 2.3)
Study Selection	9	State the process for selecting studies (i.e., screening, eligibility, included in systematic review, and, if applicable, included in the meta-analysis).	Page 6 (Section 2.4)
Data Collection Process	10	Describe method of data extraction from reports (e.g., piloted forms, independently, in duplicate) and any processes for obtaining and confirming data from investigators.	Pages 6–7 (Section 2.5)
Data Items	11	List and define all variables for which data were sought (e.g., PICO, funding sources) and any assumptions and simplifications made.	Pages 6–7 (Section 2.5)
Risk of Bias and Applicability	12	Describe methods used for assessing risk of bias and applicability of individual studies (e.g., QUADAS-2), and how this information is to be used in any data synthesis.	Page 7 (Section 2.6)
Summary Measures	13	State the principal summary measures (e.g., sensitivity, specificity, DOR).	Pages 7–8 (Section 2.7)
Synthesis of Results	14	Describe the methods of handling data and combining results of studies, including measures of consistency (e.g., $I^2$) and selection of models (e.g., bivariate, HSROC).	Pages 7–8 (Section 2.7)
Risk of Bias Across Studies	15	Describe any assessment of risk of bias across studies (e.g., publication bias).	Page 8 (Section 2.7.2)
Additional Analyses	16	Describe methods of additional analyses (e.g., sensitivity or subgroup analyses), if done, indicating which were pre-specified.	Pages 8–9 (Section 2.7.2 and Section 2.9)
RESULTS			
Study Selection	17	Give numbers of studies screened, assessed for eligibility, and included in the review, with reasons for exclusions at each stage, ideally with a flow diagram.	Pages 9–11 (Section 3.1, Figure 1)
Study Characteristics	18	For each study, present characteristics for which data were extracted (e.g., sample size, BMI cut-off, country, GDM prevalence).	Pages 9 (Section 3.2, Table 1)
Risk of Bias and Applicability	19	Present data on risk of bias and applicability of included studies.	Pages 11–12 (Section 3.3, Table 2)
Results of Individual Studies	20	For all included studies, present the 2 × 2 data (TP, FP, FN, TN) and estimated effects (sensitivity/specificity).	Pages 13–18 (Figure 2, Table 4)
Synthesis of Results	21	Present the main results of the review, including forest plots and summary estimates with confidence intervals.	Pages 12–15 (Section 3.4 and Section 3.5, Table 3)
Robustness of Synthesis	22	Describe any sensitivity analyses or assessments of heterogeneity/publication bias.	Page 16 (Section 3.6 and Section 3.7)
DISCUSSION			
Summary of Evidence	23	Summarize the main findings including the strength of evidence for each main outcome.	Pages 18–20 (Section 4.1 and Section 4.2)
Limitations	24	Discuss limitations at study and outcome level (e.g., risk of bias), and at review-level (e.g., incomplete retrieval of identified research).	Pages 22–23 (Section 4.7)
Conclusions	25	Provide a general interpretation of the results in the context of other evidence, and implications for future research and clinical practice.	Page 24 (Section 5)
FUNDING			
Funding	26	Describe sources of funding for the systematic review and other support (e.g., supply of data); role of funders for the systematic review.	Page 25 (Declarations)
